# Comparison of basic and specialized care between nursing assistants and professional nurses

**DOI:** 10.15649/cuidarte.5555

**Published:** 2026-08-06

**Authors:** Ángela Fernanda Espinosa Aranzales, Sandra Milena Tibaquirá Torres, Anacaona Martínez Del Valle, Ana Lucía Casallas Murillo, Adriana del Pilar Patiño Quintero

**Affiliations:** 1 School of Medicine and Health Sciences, Universidad del Rosario, Bogotá, Colombia. E-mail: angela.espinosa@urosario.edu.co Universidad del Rosario Bogotá Colombia angela.espinosa@urosario.edu.co; 2 Hospital Universitario Mayor – Méderi, Universidad del Rosario, Bogotá, Colombia. E-mail: sandra.tibaquira@mederi.com.co Universidad del Rosario Bogotá Colombia sandra.tibaquira@mederi.com.co; 3 Auna Academic Unit, Medellín, Colombia. E-mail: anacaona.martinez@auna.org Auna Academic Unit Medellín Colombia anacaona.martinez@auna.org; 4 School of Medicine and Health Sciences, Universidad del Rosario, Bogotá, Colombia. E-mail: ana.casallas@urosario.edu.co Universidad del Rosario Bogotá Colombia ana.casallas@urosario.edu.co; 5 Registered Nurse. Bogotá, Colombia. E-mail: adripila@icloud.com Registered Nurse Bogotá Colombia adripila@icloud.com

**Keywords:** Delegation, Professional, Nursing Care, Nurse’s Role, Nursing Assistants, Nurses, Professional Training, Delegación Profesional, Cuidado de Enfermería, Rol de la Enfermera, Auxiliares de Enfermería, Enfermeras, Capacitación Profesional, Delegação Vertical de Responsabilidades Profissionais, Cuidados de Enfermagen, Papel do Profissional de Enfermagem, Assistentes de Enfermagem, Enfermeiras; Capacitação Profissional

## Abstract

**Introduction:**

Professional nurses are trained to provide direct care; however, in hospital settings, they delegate tasks to nursing assistants, which can lead to adverse events and patient dissatisfaction.

**Objective:**

To analyze basic and specialized nursing care activities, classifying them according to the scope of competence and level of criticality.

**Materials and Methods:**

This cross-sectional observational study was conducted between June 2021 and January 2022 and included 141 professional nurses and 259 nursing assistants from a university hospital in Bogotá. A structured questionnaire was used to record the activities performed by both roles, along with their frequency and execution time. Expert consensus was established regarding the set of nursing care activities, their alignment with each role’s scope of practice, and the classification of critical activities representing a risk when delegated. Descriptive analysis identified who performed each activity, along with its frequency and execution time. Bivariate analysis assessed differences between the activities performed.

**Results:**

A total of 40 basic nursing activities and 17 specialized nursing activities were identified, of which 18 were classified as critical. Significant differences were observed between nursing assistants and professional nurses in the performance and frequency of activities, including neurological assessment, pain management, and medication monitoring.

**Discussion:**

The delegation of critical activities to nursing assistants, driven by the multiplicity of administrative and care-related tasks and by inadequate staffing of professional nurses, highlights the need to examine the negative consequences for patients.

**Conclusion:**

At the hospital evaluated, nursing assistants perform activities that, given the required level of competence and the risks involved, should be carried out by professional nurses.

## Resumen

**Introducción:**

El profesional de enfermería, formado para brindar cuidados directos; en el ámbito hospitalario delega tareas al personal auxiliar, esto puede generar eventos adversos e insatisfacción en los pacientes.

**Objetivo:**

Analizar actividades de cuidado básico y especializado realizadas por enfermería, clasificándolas según su correspondencia y criticidad conforme a la formación.

**Materiales y métodos:**

Estudio observacional transversal, que entre junio 2021 y enero 2022 incluyó a 141 profesionales y 259 auxiliares de un hospital universitario en Bogotá. A través de un cuestionario estructurado, se registraron las actividades realizadas por ambos roles, así como la frecuencia y tiempo de ejecución, se establecieron consensos de expertas, sobre el conjunto de actividades asistenciales, la correspondencia según el rol y clasificación de actividades críticas que representan un riesgo al ser delegadas. El análisis descriptivo identificó quién realizó la actividad, la frecuencia y el tiempo de ejecución. El bivariado, evaluó diferencias entre las actividades ejecutadas.

**Resultados:**

Se identificaron 40 actividades de cuidado básico y 17 de especializado, de ellas 18 se consideraron críticas. Hubo diferencias significativas entre auxiliares y profesionales en la ejecución y frecuencia de actividades como valoración neurológica, manejo del dolor y monitoreo farmacológico.

**Discusión:**

Delegar actividades críticas al personal auxiliar por la multiplicidad de tareas y la escasa contratación de profesionales conduce a examinar circunstancias negativas para los pacientes.

**Conclusiones:**

En el hospital evaluado, el personal auxiliar realiza actividades que por su nivel de formación y por el riesgo que suponen corresponderían, al profesional de enfermería.

## Resumo

**Introdução:**

Os profissionais de enfermagem são treinados para prestar cuidados diretos; eles delegam tarefas à equipe auxiliar no ambiente hospitalar. Isso pode levar a eventos adversos e insatisfação do paciente.

**Objetivo:**

Analisar as atividades de cuidado básico e especializado realizadas por enfermeiros, classificando-as de acordo com sua relevância e criticidade com base em sua formação.

**Materiais e métodos:**

Foi realizado um estudo observacional transversal entre junho de 2021 e janeiro de 2022, incluindo 141 enfermeiros e 259 auxiliares de enfermagem de um hospital universitário em Bogotá. Um questionário estruturado foi utilizado para registrar as atividades realizadas por ambos os grupos, bem como sua frequência e tempo de execução. O consenso entre especialistas foi estabelecido em relação ao conjunto de atividades de cuidado, sua relevância para cada grupo e a classificação das atividades críticas que representam um risco quando delegadas. A análise descritiva identificou quem realizou cada atividade, sua frequência e tempo de execução. A análise bivariada avaliou as diferenças entre as atividades realizadas.

**Resultados:**

Foram identificadas 40 atividades de cuidado básico e 17 atividades de cuidado especializado; Dezoito dessas atividades foram consideradas críticas. Foram encontradas diferenças significativas entre auxiliares de enfermagem e profissionais de enfermagem na execução e frequência de atividades como avaliação neurológica, manejo da dor e monitoramento farmacológico.

**Discussão:**

Delegar atividades críticas a auxiliares de enfermagem devido à multiplicidade de tarefas e à contratação limitada de profissionais de enfermagem leva a consequências negativas para os pacientes.

**Conclusões:**

No hospital avaliado, os auxiliares de enfermagem realizam atividades que, dado seu nível de formação e os riscos envolvidos, deveriam ser executadas por profissionais de enfermagem.

## Introduction

In hospital settings, direct care provided by professional nurses is essential for achieving better health outcomes^1^; showever, the nursing workforce is currently insufficient to meet care demands^2^. This situation leads to the delegation of professional nursing activities to nursing assistants^3^. These practices should be examined more thoroughly to determine which activities can or cannot be delegated, considering education and competencies, in order to ensure care quality and safety^4^,^5^. Task, circumstance, person, directions and communication, and supervision and evaluation are the five principles of appropriate delegation^6^; however, inadequate delegation may lead to adverse events, delays in care, and patient dissatisfaction^7^,^8^.

In the United States and Canada, nursing delegation is regulated by national guidelines^9^,^10^ that distinguish three categories of healthcare personnel: Registered nurses (RN), professional nurses with 4 years of university education focused on clinical reasoning, planning, supervision and administration^11^; licensed practical nurses/licensed vocational nurses (LPN/LVN), who complete 12- to 18-month technical education programs in the United States^11^, and 2- to 3- year post secondary technical education programs in Canada^12^; and are oriented toward basic and intermediate patient care, with more specific competencies than nursing assistants and partial autonomy, as they work under RN supervision^11^. Finally, unlicensed assistive personnel or healthcare assistants (UAP and HCA), whose education ranges from a few weeks to 6 months and is not formally regulated in Canada, are focused on basic patient care and always work under RN supervision. In these countries, the registered nurse (RN) remains responsible for the delegated tasks, which are delegated only when the delegatee demonstrates competence and the tasks involve low clinical risk^10^,^11^.

In Brazil, the Conselho Federal de Enfermagem (COFEN)^13^ establishes three categories of nursing personnel: nurses, with 4-5 years of university education focused on comprehensive care, planning, and supervision; nursing technicians, with 2 years of postsecondary technical education focused on intermediate care and specific procedures under nurse supervision; and nursing assistants, with one year of secondary-level education oriented toward basic patient care^13^. In Colombia, the nursing education and service delivery model is binary—that is, professional nurses and nursing assistants—and there is no formally validated national system of delegable responsibilities^14^-^16^. Other countries have their own defined models of practice and task delegation^9^,^10^.

In Colombia, undergraduate nursing education lasts between 4 and 5 years, and its scope of practice includes basic and specialized nursing care grounded in a comprehensive view of the care recipient^15^, autonomy^14^ and the promotion of human development^16^,^17^. Nursing assistants complete 1 to 2 years of secondary-level technical education focused on basic comprehensive patient care^18^ and perform their duties under the direct supervision of healthcare professionals^19^,^20^. Therefore, professional nurses may delegate activities to nursing assistants when, based on an assessment of factors such as time, setting, and person, delegation is considered appropriate^14^,^21^.

Given the lack of formal mechanisms for delegation, it is concerning that nursing assistants in Colombia assume responsibilities beyond their competence^22^,^23^. Furthermore, few studies have examined in depth the role of nursing personnel, the largest workforce in the healthcare sector^16^. In tertiary care hospitals, the distribution of nursing personnel varies by service type, whether basic or specialized^24^. Professional nurses devote time to administrative tasks^25^,^26^ and delegate direct patient care, increasing the probability of adverse events and diminishing the visibility of their work within the healthcare system and society at large^16^,^27^.

Basic nursing care focuses on meeting patients’ basic daily needs, such as hygiene, feeding, mobility, and repositioning, and falls within the scope of practice of nursing assistants. In contrast, specialized nursing care involves more complex activities requiring clinical judgment and advanced education, such as vascular access procedures, catheterization, wound care, and medication administration, which fall within the scope of practice of professional nurses^28^,^29^. Direct care provided by professional nurses promotes interpersonal relationships and enhances communication within the therapeutic process. These practices are based on nursing models and on the Nursing Interventions Classification (NIC)^30^.

This study analyzed basic and specialized nursing activities performed by nursing assistants that fall outside their scope of practice. To address this issue, the guiding research question was as follows: Which activities performed by nursing assistants in a university hospital in Bogotá fall outside their scope of practice, given their competence level? Addressing this question represents an initial step toward organizing the distribution of role-specific activities and may positively affect humanized patient care, healthcare team functioning, and nursing documentation.

## Materials and Methods

A cross-sectional observational study was conducted. Participants included nursing assistants with 18 months of vocational education and a technical qualification as a nursing assistant, as well as professional nurses with four to five years of university education and a formal nursing degree. Both groups provided patient care during morning, afternoon, full-day, or night shifts at two sites of a tertiary care university hospital located in Bogotá, Colombia. The nursing population at the study hospital consisted of 740 nursing assistants and 276 professional nurses. The inclusion criterion was active involvement in direct patient care. The sample size calculation was based on the hospital’s total nursing staff population, and the different hospital services were weighted by the number of nursing assistants and professional nurses assigned to each. A prevalence of role-specific activities based on uncertainty (p)^31^ was used, employing the statistical software Epi Info, version 7.2.2.6. Finally, a random sample of 161 professional nurses and 253 nursing assistants was determined to estimate a population event proportion of 50% at the 95% confidence level and 80% statistical power.

**Data collection**

During meetings with nursing staff, the nursing department provided the research team with time to invite all personnel to participate in the study. Participant enrollment was consecutive until the target sample size was reached. Data were collected between June 2021 and January 2022 using a self-administered survey or a survey administered by trained research assistants. Surveys were completed at the end of the shift to facilitate recall and took an average of 25 minutes.

**Measurements**

Based on the Nursing Interventions Classification (NIC)^30^ and the protocols of the institution where the study was conducted, the researchers developed a questionnaire to assess the activities performed during each shift. The instrument collected sociodemographic information, such as age, sex, and education; occupational variables, including shift, service, role performed, clinical experience, and length of employment at the institution; and variables related to the research topic, including the activity performed, its frequency, and the time required for its completion.

The researchers reached consensus on the activities performed by nursing personnel during each shift, defining five categories: basic nursing care, specialized nursing care, education and communication, management, and other activities not directly related to patient care but assigned to nursing personnel. An Excel matrix was used with the activities listed in the first column and the five categories in the subsequent columns. Each activity was classified into a category when at least 75% of the group reached agreement. This manuscript presents information on basic and specialized nursing care.

**Figure 1:**
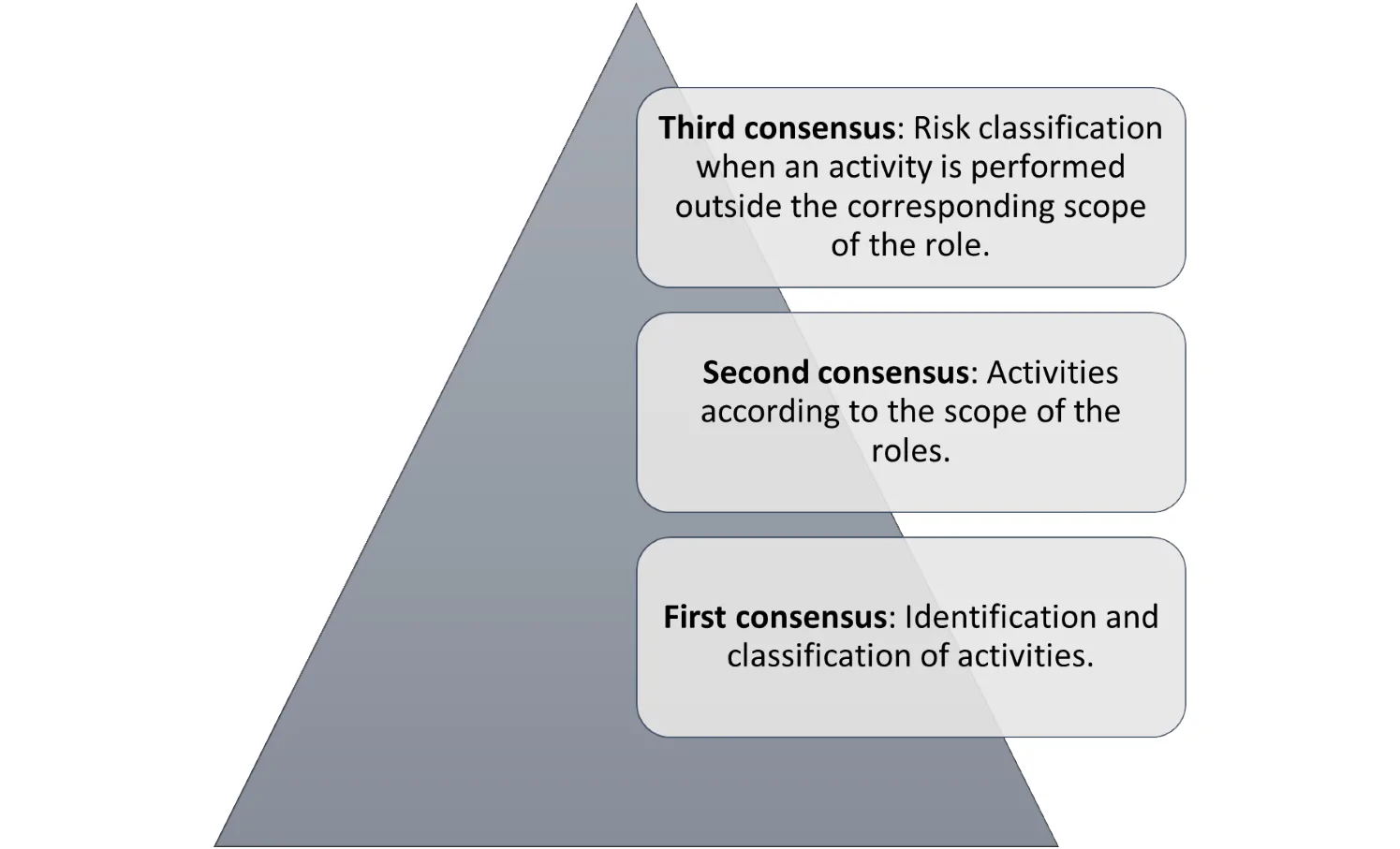
Representation of the three consensus processes conducted in the study

To determine whether an activity fell within the scope of practice of nursing assistants or professional nurses, a second consensus process was conducted using the same procedure described above. The same approach was used to identify risk or critical situations in which an activity performed by a nursing assistant fell within the scope of practice of a professional nurse because it required specialized competence or involved a higher level of complexity Figure 1.

Basic nursing care corresponded to activities aimed at providing patient comfort and safety, as well as procedures such as medication administration, peripheral vascular access, nasogastric or orogastric intubation, urinary catheterization, and neurological assessment. Specialized nursing care included activities performed by nursing staff for patients or services requiring greater complexity of care, such as fetal monitoring, chemotherapy administration, and post-anesthesia care^32^.

Hospitalization, emergency, internal medicine, and gynecology services were classified as general services, whereas operating rooms, delivery rooms, intensive care units, neonatology, the kangaroo care program, and oncology were classified as specialized services. Regarding shift duration, morning and afternoon shifts lasted 6.5 hours, whereas night shifts lasted 12.5 hours.

**Data analysis**

Sociodemographic variables, occupational variables, and activities were described using absolute and relative frequencies, medians, and minimum and maximum values, depending on the variable type and distribution. Based on the consensus regarding role-specific activities, activities classified as critical in each category were selected to estimate whether statistically significant differences existed between nursing assistants and professional nurses regarding the number of responses indicating performance of the activity, using the chi-square test or Fisher’s exact test, as appropriate, and the median frequency and duration of these activities using the Mann-Whitney test. Calculations of median frequency and time were based on the maximum values, as these were considered to represent the extreme values for both activity frequency and duration. All collected data are freely available for access and consultation through Mendeley Data^33^.

**Ethical considerations**

The research project was approved by the Institutional Research and Ethics Committee of Universidad del Rosario, approval number DVO005 983-CV1165. All the participants provided signed informed consent.

## Results

A total of 400 individuals participated in the study, including 259 nursing assistants and 141 professional nurses. Most participants were female, between 18 and 35 years of age, and had educational backgrounds consistent with their roles, except for two respondents with nursing degrees who were working as nursing assistants. Most participants worked morning shifts in non-specialized services, and nursing assistants had longer clinical experience and longer tenure at the institution Table 1.

**Table 1:** Participants’ socio-demographic characteristics: Qualitative variables

Variables	Survey	
	Nursing assistants n=259 % (n)	Professional nurses n=141 % (n)
Sex		
Female	83.78 (217)	80.85 (114)
Male	16.21 (42)	19.14 (27)
Age		
18 to 35 years	63.32 (164)	65.95 (93)
36 to 55 years	32.43 (84)	32.62 (46)
56 to 65 years	4.24 (11)	1.41 (2)
Educational level		
Postgraduate degree	0 (0)	13.47 (19)
Nursing degree	0.77 (2)	86.52 (122)
Technical qualification	99.22 (257)	0 (0)
Shift		
Morning	37.45 (97)	29.78 (42)
Afternoon	27.41 (71)	35.46 (50)
Full daytime shift	3.86 (10)	4.96 (7)
Night	31.27 (81)	29.78 (42)
Services		
General	57.91 (150)	64.53 (91)
Specialized	42.08 (109)	35.46 (50)

**Identification of activities**

In basic nursing care, a total of 40 activities were identified, of which 17 fell within the scope of practice of professional nurses, 18 within that of nursing assistants, and 5 were shared by both roles. In specialized nursing care, a total of 17 activities were identified: 10 within the scope of practice of professional nurses, 5 within that of nursing assistants, and 2 shared by both roles.

**Role-specific activities**

**Basic nursing activities**

Among the 259 nursing assistants, 37.45% reported performing neurological assessments, compared with 26% of the 141 professional nurses, a statistically significant difference (p= 0.02). However, no statistically significant differences were observed in the frequency or time spent performing this activity between roles. Nursing care for pain management was performed by 24.71% of the nursing assistants and 34% of the professional nurses (p= 0.04). When evaluating the frequency of activity performance, nursing assistants reported monitoring medication effects a median of 9 times, whereas professional nurses reported performing this activity 4 times (p= 0.00). Medication administration was the most time-consuming activity among professional nurses, whereas specialized specimen collection for testing required the most time among nursing assistants Table 2.

**Table 2:** Critical basic nursing activities reported in the survey

Activity	Activity performed during the shift Yes			Frequency			Time in minutes		
	Nursing assistant n:259 % (n)	Professional nurse n:141 % (n)	p-value	Nursing assistant n:259 Med [IQR]	Professional nurse n:141 Med [IQR]	p-value	Nursing assistant n:259 Med [IQR]	Professional nurse n:141 Med [IQR]	p-value
Medication administration	5.79 (15)	80.14 (113)	0.01	7[3;22]	3[2;4]	0.03	6[3;10]	60[30;90]	0.01
Monitoring of medication effects	5.41 (14)	41.13 (58)	0.01	9[4;30]	4[3;6]	0.01	8[2;10]	14[9;38]	0.02
Parenteral nutrition administration	5.41 (14)	8.51 (12)	0.22	1[1;2]	3[1;5]	0.38	4[2;6]	10[10;15]	0.01
Drainage Care	13.90 (36)	8.51 (12)	0.11	2[1;3]	2[2;3]	0.51	5[3;10]	10[10;15]	0.01
Nasogastric intubation	4.25 (11)	10.64 (15)	0.01	2[1;2]	2[2;3]	0.51	15[8;18]	15[14;28]	0.76
Orogastric intubation	3.09 (8)	7.80 (11)	0.03	2[2;2]	2[2;3]	0.65	15[6;16]	15[11;23]	0.49
Specialized specimen collection and blood cultures	23.2 (6)	19.15 (27)	0.01	2[1;3]	2[2;3]	0.42	23[16;29]	35[18;60]	0.12
Nursing care for pain management	24.71 (64)	34.04(48)	0.04	6[3;10]	6[3;17]	0.40	5[3;10]	10[5;13]	0.01
Neurological assessment	37.45 (97)	26.24(37)	0.02	8[3;10]	8[3;17]	0.27	4[2;10]	10[3;15]	0.01

Med: Median; IQR: Interquartile range; The tests used to evaluate differences between assistants and professionals regarding whether or not they performed the activity were the Chi-square and Fisher's exact tests, and the test used to evaluate differences regarding frequency and execution time was the Mann-Whitney test.

**Specialized nursing activities**

Both nursing assistants and professional nurses reported performing the 9 critical activities. Statistically significant differences between roles were observed in reporting, frequency, and time devoted to injection administration, specialized services, assessment of diagnostic test results, and clinical documentation Table 3.

**Table 3:** Specialized nursing activities reported in the survey

Activity	Number of participants			Frequency			Time in minutes		
	Nursing Assistant n:259 %(n)	Professional nurse n:141 %(n)	p-value	Nursing Assistant n:259 Med [IQR]	Professional nurse n:141 Med [IQR]	p-value	Nursing Assistant n:259 Med [IQR]	Professional nurse n:141 Med [IQR]	p-value
Fetal monitoring interpretation	1.93 (5)	0.71 (1)	0.33	5[2;6]	8[8;8]	0.67	25[20;25]	25[25;25]	0.67
Management of fetal complications	0.77 (2)	1.42 (2)	0.53	4[4;4]	13[10;16]	0.67	9[8.5;9.5]	17.5[14;21]	0.33
Postpartum hemorrhage assessment and management	1.16 (3)	3.55 (5)	0.10	3.50[2;6]	7[5.5;12]	0.40	20[18;20]	17[15;20]	0.57
Post-anesthesia care	2.32 (6)	2.84 (4)	0.75	5[3.5;7]	2[1.5;3.5]	0.27	10[6;14]	17.5[12;195]	0.35
Chemotherapy administration and monitoring	0.77 (2)	3.55 (5)	0.04	5.50[4.8;6.2]	5[4;6]	0.86	4[3.5;4.5]	40[20;360]	0.19
Hemodynamic monitoring and interpretation	5.02 (13)	14.89 (21)	0.01	9[3;13]	5[5;17]	0.33	10[10;15]	10[10;15]	0.94
Intramuscular and intravenous medication administration in specialized services.	10.81 (28)	24.11 (34)	0.01	4[2;8.5]	10[4.25;18]	0.02	3[2;5.25]	15[8;30]	0.01
Blood product administration	5.41 (14)	21.28 (30)	0.01	3[1;4.25]	2[2;4]	0.82	105[33.8;120]	120[40;120]	0.48
Diagnostic test assessment and clinical documentation	2.70 (7)	29.79(42)	0.01	2[1.5;2.5]	6[5;10]	0.01	4[1.5;4.5]	15[8;30]	0.01

Med: Median; IQR: Interquartile Range; The tests used to evaluate differences between assistants and professionals regarding whether or not they performed the activity were the Chi-square and Fisher's exact tests, and the test used to evaluate differences regarding frequency and execution time was the Mann-Whitney test.

## Discussion

Within the categories of basic and specialized nursing care, activities classified as critical were identified when performed by nursing assistants. Nursing assistants showed greater participation in basic nursing activities, whereas professional nurses showed greater participation in specialized nursing care activities.

The researchers defined three lines of analysis regarding activities performed by nursing assistants that fall outside their role. The first concerns institutional factors that determine nursing practice; the second concerns the delegation of activities from professional nurses to nursing assistants in response to workload demands, both of which have potential effects on patient health outcomes; and the third concerns activities labeled identically regardless of the role performing them. These lines of analysis are further discussed below.

The nursing assistants’ education in Colombia is oriented toward basic patient care; however, findings from the study institution indicate that personnel in this role perform specialized nursing activities that require clinical judgment and advanced education. Thus, a mismatch between competence and professional practice is observed. In countries such as the United States, Canada, and Brazil, formal national guidelines and/or mechanisms exist regarding the competencies and defined limitations of personnel at each level of care, including professional nurses, nursing technicians, and nursing assistants^9^,^10^,^13^. The official definition of the competencies and responsibilities of each group, along with the existence of an intermediate level of care focused on basic care and some specialized care activities, differs from what was identified at the institution where the present study was conducted.

Nursing assistants participate in medication administration and parenteral nutrition administration, although institutional protocols define these activities within the scope of practice of professional nurses. Nevertheless, it is established that nursing assistants may exceptionally perform these activities in high-volume clinical services. Authors such as Maghsoud et al.^34^ noted that workload leads to task delegation and negatively affects the quality of nursing care in specialized services. Wilson et al.^35^ reported that delegation to nursing assistants or staff without formal education may lead to consequences such as medication errors, incomplete clinical documentation, lower patient satisfaction, and confusion regarding professional roles. Despite mentioning the positive effects of delegating tasks, such as improved efficiency in high-volume clinical services, Shore et al.^36^ noted that these benefits are primarily linked to adequate competence and delegation conducted under clear institutional regulations.

In addition, medication administration by nursing assistants results from professional nurses delegating to them in response to a high workload^37^. In 2023, Kagonya et al.^38^ identified medication administration as an activity performed by nursing assistants. Similarly, Fitzgerald et al.^39^, when evaluating the role of healthcare assistants, describe how professional nurses, due to high workload, delegate time-consuming tasks to untrained personnel, particularly those considered low in complexity. One possible explanation for why nursing assistants in Colombia perform this activity is that the nurse-to-patient ratio falls below international standards^16^,^31^,^32^,^34^,^37^–^40^. Studies conducted in the United States^41^,^42^ and Canada^43^ have reported positive effects of task delegation to assistive personnel, including greater patient and staff satisfaction and increased efficiency, when competencies are validated within a formal national system for nursing delegation.

Furthermore, the eight activities described below are delegated to nursing assistants and fall within their role, including drainage care and pain management. However, during drainage care, nursing assistants monitor drainage output while also providing care instructions and, in pain management, use basic comfort assessment scales. These activities are intended for professional nurses, particularly patient education on self-care and the early identification of complications. The literature reports that, due to workload demands, professional nurses omit role-specific activities, a phenomenon known as missed nursing care, which may affect patient safety^44^–^46^.

Nasogastric and orogastric intubations are activities performed by nursing assistants in response to high demands placed on professional nurses. However, due to adverse events, the institution assigns these procedures to professional nurses. Kim et al.^47^ found poorer health outcomes in services where direct care was provided primarily by nursing assistants or where an appropriate nurse-to-patient ratio was lacking.

Nursing assistants participate in neurological assessment, fetal monitoring interpretation, hemodynamic monitoring, assessment and management of postpartum hemorrhage, post-anesthesia care, and monitoring medication effects. Although nursing assistants may participate in the assessment stage of these activities, interpretation and decision-making fall within the scope of practice of professional nurses because alterations may be underestimated.

In addition, no differences were observed between the two roles in the management of fetal complications, suggesting that nursing assistants intervene only when they identify an abnormality, which may affect the quality of care. Although the interpretation of diagnostic test results and the clinical documentation are activities exclusive to professional nurses, the study identified participation of nursing assistants in these activities, with the risk of failing to identify potential complications in patient management promptly. Duffield et al.^32^, in a study evaluating health outcomes associated with the participation of nursing assistants, emphasized the importance of distinguishing between basic tasks and those requiring decision-making to achieve better health outcomes.

In the United States, the national guidelines for nursing delegation establish five rights of delegation: right task, right person, right circumstance, right directions and communication, and right supervision and evaluation^9^. In the study institution, according to these guidelines, tasks that fall outside the scope of practice of nursing assistants are delegated. This research did not examine the remaining components.

Blood product and chemotherapy administration fall exclusively within the scope of practice of professional nurses; the participation reported by nursing assistants may be attributed to a supportive role during their execution. Thus, this participation is assumed to be complementary rather than substitutive, based on differences observed in frequency and duration. Similarly, the participation of nursing assistants in specialized specimen collection and blood cultures may be attributed to overlap in the conceptualization of the activity or to poorly defined role boundaries. In this regard, the Nursing Interventions Classification (NIC)^30^ is not standardized according to professional role, which may lead to overlap in institutional protocols.

There are other ways of organizing nursing care. For example, Dubois et al.^48^ describe four role-based nursing care models that optimize the workforce distribution. Although workload and nurse-to-patient ratios lead to task delegation and may necessitate adjustments, it is necessary to assess their implications for the quality, safety, and timeliness of meeting patient needs^49^.

The cost-reduction perspective and the limited availability of professional nurses tend to normalize the delegation of professional nursing tasks to nursing assistants, without considering the impact on patient care^50^.

Policies aimed at strengthening nursing practice should consider circumstances such as time, setting, and expected health outcomes^51^. As demonstrated by this study, it is essential to define, within job descriptions, the activities assigned to each role, establish their scope of practice, specify responsibilities, and determine the conditions under which tasks may be delegated.

Finally, study limitations to consider include the time gap between the invitation to participate and the start of fieldwork due to the COVID-19 pandemic, as well as changes in service assignment among some participants, which initially hindered implementation of the weighted sampling plan. Nevertheless, a greater proportion of participants from general services was achieved. It should also be noted that during the consensus process for identifying activities, it was not possible to clearly distinguish the activities performed by each role for some tasks, making it difficult to estimate role-specific activities.

Likewise, different stages of the COVID-19 pandemic occurred in Bogotá during the study period, including successive waves of increased and decreased transmission^52^-^54^, which may have influenced the study results and favored the delegation of specialized tasks to nursing assistants.

## Conclusions

The study confirms that nursing assistants perform activities beyond their scope of competence. This situation arises from delegation practices adopted by institutions as an alternative to address the shortage of professional nurses, as well as from the lack of clear differentiation in institutional protocols regarding the responsibilities assigned to each role. Delegation is also promoted by professional nurses in response to a high workload. Both factors may negatively affect patient safety and contribute to missed nursing care and compromised quality of care. The widespread normalization of delegating activities inherent to professional nursing competencies affects patients and their families, poses a risk to healthcare institutions through the performance of procedures that may compromise patient safety, and ultimately affects the nursing profession as a whole by blurring its identity, its social role in care delivery and healthcare services, and reducing recognition of its professional profile while contributing to precarious working conditions.

This study contributes to the analysis of basic and specialized clinical care activities performed by both professional nurses and nursing assistants, highlighting the limitations of national regulations and guidelines regarding the scope of practice for each role, a situation that ultimately determines the quality of care actions.
